# The clinical complexity of cases of schizophrenia in a community mental health team: a 3 year comparison

**DOI:** 10.1192/bjo.2021.892

**Published:** 2021-06-18

**Authors:** Shay-Anne Pantall, Laxsan Karunanithy, Hayley Boden, Lisa Brownell

**Affiliations:** 1 Birmingham and Solihull Mental Health NHS Foundation Trust; 2 University of Birmingham, Medical School

## Abstract

**Aims:**

To describe the changes in complexity and management of individuals with schizophrenia in a community mental health team (CMHT) over a three year period.

**Background:**

It is often believed that individuals receiving care from CMHTs are those with low levels of complexity and risk, and are relatively stable, with more complex individuals being managed by assertive outreach or other specialist teams. Here, we describe changes in the complexity, comorbidity, service-usage and management, of patients with a diagnosis of schizophrenia in a CMHT between 2016 and 2019.

**Method:**

Data were collected from an electronic patient record system (RiO) for all individuals with schizophrenia in a CMHT in Birmingham (n = 84 in 2016, n = 71 in 2019), examining demographic variables, comorbidity, use of mental health services and current management.

**Result:**

Key findings included: -

- 63% were managed through care programme approach (CPA) in 2016, compared to only 31% in 2019.
- 21% had required home treatment or inpatient care in the preceding 12 months in 2016; this had improved to 8.5% in 2019.
- Significant levels of psychiatric comorbidity, including addictions with almost half of patients (46.5%) having a known history of substance use in 2019, compared to only 15.5% noted in 2016.
- Pharmacological management has remained broadly similar; in 2016 21% patients were taking a combination of 2 antipsychotics compared to only 10% in 2019 and 25% were taking clozapine in 2016 (21% in 2019). 39% were prescribed a long acting antipsychotic injection in 2016, compared to 32% in 2019.
- In 2016, medication was being prescribed in the majority of cases within secondary care (55%) patients and in primary care in only 21%. GPs have now taken on greater prescribing responsibility in 2019, prescribing in 44% of cases, with 47% being prescribed by the CMHT.

**Conclusion:**

The acuity and management of individuals with a diagnosis of schizophrenia under the care of a CMHT has changed over a 3 year period. It is positive to note the reduced use of crisis services and lower rates of polypharmacy. There is a reduction in the proportion of patients receiving management through CPA, and a move towards more medication being prescribed in primary care. The reasons for this change are however unclear and may reflect change in available resources, given that more than half of this group receive clozapine or long acting injections, and have high levels of comorbidity.

